# Linguistic Methodologies to Surveil the Leading Causes of Mortality: Scoping Review of Twitter for Public Health Data

**DOI:** 10.2196/39484

**Published:** 2023-06-12

**Authors:** Jamil M Lane, Daniel Habib, Brenda Curtis

**Affiliations:** 1 Department of Environmental Medicine and Public Health Icahn School of Medicine at Mount Sinai New York, NY United States; 2 Technology and Translational Research Unit National Institute on Drug Abuse National Institutes of Health Baltimore, MD United States

**Keywords:** Twitter, public health interventions, surveillance data, health communication, natural language processing

## Abstract

**Background:**

Twitter has become a dominant source of public health data and a widely used method to investigate and understand public health–related issues internationally. By leveraging big data methodologies to mine Twitter for health-related data at the individual and community levels, scientists can use the data as a rapid and less expensive source for both epidemiological surveillance and studies on human behavior. However, limited reviews have focused on novel applications of language analyses that examine human health and behavior and the surveillance of several emerging diseases, chronic conditions, and risky behaviors.

**Objective:**

The primary focus of this scoping review was to provide a comprehensive overview of relevant studies that have used Twitter as a data source in public health research to analyze users’ tweets to identify and understand physical and mental health conditions and remotely monitor the leading causes of mortality related to emerging disease epidemics, chronic diseases, and risk behaviors.

**Methods:**

A literature search strategy following the PRISMA (Preferred Reporting Items for Systematic Reviews and Meta-Analyses) extended guidelines for scoping reviews was used to search specific keywords on Twitter and public health on 5 databases: Web of Science, PubMed, CINAHL, PsycINFO, and Google Scholar. We reviewed the literature comprising peer-reviewed empirical research articles that included original research published in English-language journals between 2008 and 2021. Key information on Twitter data being leveraged for analyzing user language to study physical and mental health and public health surveillance was extracted.

**Results:**

A total of 38 articles that focused primarily on Twitter as a data source met the inclusion criteria for review. In total, two themes emerged from the literature: (1) language analysis to identify health threats and physical and mental health understandings about people and societies and (2) public health surveillance related to leading causes of mortality, primarily representing 3 categories (ie, respiratory infections, cardiovascular disease, and COVID-19). The findings suggest that Twitter language data can be mined to detect mental health conditions, disease surveillance, and death rates; identify heart-related content; show how health-related information is shared and discussed; and provide access to users’ opinions and feelings.

**Conclusions:**

Twitter analysis shows promise in the field of public health communication and surveillance. It may be essential to use Twitter to supplement more conventional public health surveillance approaches. Twitter can potentially fortify researchers’ ability to collect data in a timely way and improve the early identification of potential health threats. Twitter can also help identify subtle signals in language for understanding physical and mental health conditions.

## Introduction

### Background

Tens of millions of words, pictures, and videos are recorded on the internet via social media every day by people worldwide [1]. Some of the most popular social media platforms, such as Facebook, Instagram, Twitter, and Snapchat [2], enable social media users to interact in various innovative ways, such as sharing pictures, videos, and live streams (ie, real-time web-based video streams allowing participant interaction) [3]. Social media platforms such as Twitter have accumulated immense amounts of linguistic data from their users, with which researchers can identify patterns in individual qualities, characteristics, and social practices. Twitter is a popular web-based social networking and bulletin platform used by millions of people and organizations to post and discover information while interacting with messages known as *tweets* [4]. Twitter activity is initiated and extended by posting tweets, reposting important messages (ie, retweeting), and attracting the attention of other Twitter users (ie, followers) to an account [5]. Each day, approximately 500 million tweets are posted, conveying a diversity of topics and views [6] from >300 million active accounts worldwide [4,5]. This information, in turn, can help detect human behaviors and predict related medical conditions [1,7].

In the last decade, the internet and mobile technologies have become prevalent with increasing social media use by teenagers and adults each day [6]. In a recent study, almost 90% of 1060 adolescents aged between 13 and 17 years reported using social media, with >70% of them having a profile on multiple platforms [8]. Approximately two-thirds (65%) of adults report daily use of at least one social media platform [9]. Owing to their popularity, social media platforms represent an emerging innovative way to provide valuable information about the lives of individuals, including their health information [6]. According to Coppersmith et al [10], social media is a channel that showcases people’s language and behavior in an unbiased form, enabling researchers to diagnose certain conditions effectively.

Public health researchers use big data methodologies to mine Twitter for health-related data [4] at the individual and community levels as the platform offers unrestricted access to public tweets, unlike Facebook and Google Plus data, which are restricted and proprietary [11]. Researchers have analyzed Twitter data to gain an understanding of health-related conditions and used the data as an epidemiological surveillance source [12]. Unlike traditional surveys and tracking networks, which can be expensive and take extended periods to deliver salient information, Twitter can be less expensive for assessing health concerns and provide a faster response to and an in-depth understanding of public health threats [12,13].

### Objectives

Twitter users provide a foundation for new information by openly sharing user-generated, real-time information that is not available to their health care clinicians [12], for example, the unapproved use of medications [14]. Twitter may be a reliable and useful social media platform for public health researchers to remotely monitor vexing public health issues associated with risk behaviors and disease epidemics [15]. It may also be useful in providing potentially valuable insights for researchers who can harness Twitter’s internet-based features to provide health communication and promotion campaigns [16]. In recent years, there have been several review articles published on the benefits of using Twitter as a data source [17,18]; little work has focused on the novel applications of statistical language analyses used to study human health and behavior or on a comprehensive review on public health surveillance of leading causes of mortality related to emerging disease epidemics, chronic diseases, and risk behaviors. Therefore, the primary focus of this scoping review was to comprehensively review the literature that leverages Twitter as a source in public health research for analyzing users’ language to conduct public health surveillance of high-mortality diseases and study physical and mental health conditions. Thus, this scoping review will (1) outline the inclusion and exclusion criteria for identifying and selecting the literature that will be discussed; (2) provide a review of the literature on the 2 themes that emerged supporting Twitter being used as a public health data source; and (3) conclude with an overview of the efficacy of Twitter data and discuss the limitations, implications, and needs regarding future research in this area.

## Methods

### Overview

A scoping review methodology was used to map relevant research that has leveraged Twitter as a data source in public health research to analyze, determine, and monitor a variety of public health concerns. Using the definition presented by Arksey and O’Malley [19], a scoping review aims to “rapidly” map fundamental conceptual aspects and foundations in the relevant area and identify the primary sources, evidence, and research gaps in a comprehensive format. As Twitter has become widely used as a data source, we decided to focus on comprehensively identifying the relevant research area and summarizing and reporting research findings as per the first and third goals of a scoping review by Arksey and O’Malley [19].

### Search Strategy

A literature search strategy following the PRISMA (Preferred Reporting Items for Systematic Reviews and Meta-Analyses) extended guidelines for scoping reviews was used to search specific keywords (Textbox 1) on Twitter and public health on 5 databases: Web of Science, PubMed, CINAHL, PsycINFO, and Google Scholar. In our research, we selected commonly used search terms based on a thorough review of the literature and related studies in the field. These terms were then used as the basis for our search without expanding them to minimize the risk of bias and ensure the accuracy of the results. By carefully selecting and focusing on specific terms, we were able to limit the volume of articles retrieved, effectively narrow down the search results, and ensure that the articles obtained were relevant and of high quality. Furthermore, to ensure the comprehensive nature of our review, we thoroughly examined multiple reference lists [17,18,20] to identify any relevant articles that may have been missed in our initial search strategy. This process of checking reference lists helped ensure that all relevant studies were included in our review. In addition, we evaluated and determined that any articles not identified by our initial search strategy would not considerably affect the conclusions drawn from the review based on identifying a substantial amount (N=4986) of relevant studies.

**Key search terms.**

**Search strings**

- (“Cancer, public health, and Twitter,”); (“Twitter and COVID-19,”); (“influenza and Twitter,”); (“substance abuse and Twitter,”); (“social media language and health outcomes,”); (“Twitter language”); (“Twitter and mental health,”); (“Twitter and health data,”); (“Twitter and disease surveillance,”); (“Twitter and public health,”); (“Twitter and depression”); (“Twitter and psychology”); (“infectious Disease, surveillance, and Twitter”); (“heart disease, cardiovascular, and Twitter”); (“Twitter and physical health”)

### Study Selection

The search strategy was performed to identify relevant articles that met the following criteria: (1) peer-reviewed empirical articles representing original research from 2008 to 2021, (2) provision of research methodology and findings, (3) use of Twitter to answer research questions, and (4) being available in English (Textbox 2). We defined public health research as research that contributes to the definition of public health used by Winslow [21]: “the science and the art of preventing disease, prolonging life, and promoting health through the organized efforts and informed choices of society, organizations, public and private communities, and individuals.” The research articles were evaluated based on 4 quality criteria: the presence of a precisely defined purpose, description of techniques for mining Twitter data, discussion of methodology, and discussion of limitations [4]. After a database search leading to the identification of 4986 articles, all articles were screened by the first author, which resulted in the exclusion of 3410 (68.39%) articles screened by keywords, title and abstract, and inclusion criteria. The remaining 1576 articles were screened at the full text level to determine inclusion, resulting in the exclusion of 1295 (82.17%) articles. Of the remaining 281 articles, 38 (13.5%) were reviewed by the first 2 authors, followed by an agreed consensus on the quality inclusion criteria, and were subsequently included in the scoping review.

**Scoping review inclusion and exclusion criteria.**

**Inclusion criteria**

- Peer-reviewed empirical articles representing original research
- Published between 2008 and 2021
- Reporting research methodology and findings
- Use of Twitter data to answer research questions
- Description of techniques used for mining Twitter data
- Reporting on relevant limitations
- Available in English

**Exclusion criteria**

- Not peer-reviewed empirical articles representing original research
- Not published between 2008 and 2021
- Not reporting research methodology and findings
- No use of Twitter data to answer research questions
- No description of techniques used for mining Twitter data
- Not reporting on relevant limitations
- Not available in English

### Data Extraction

For each article, data were extracted related to (1) author, year, and location of study; (2) study objective; (3) study design; (4) study methods; (5) sample size; and (6) a succinct observation of the limitations and implications.

## Results

### Overview

**Figure 1:**
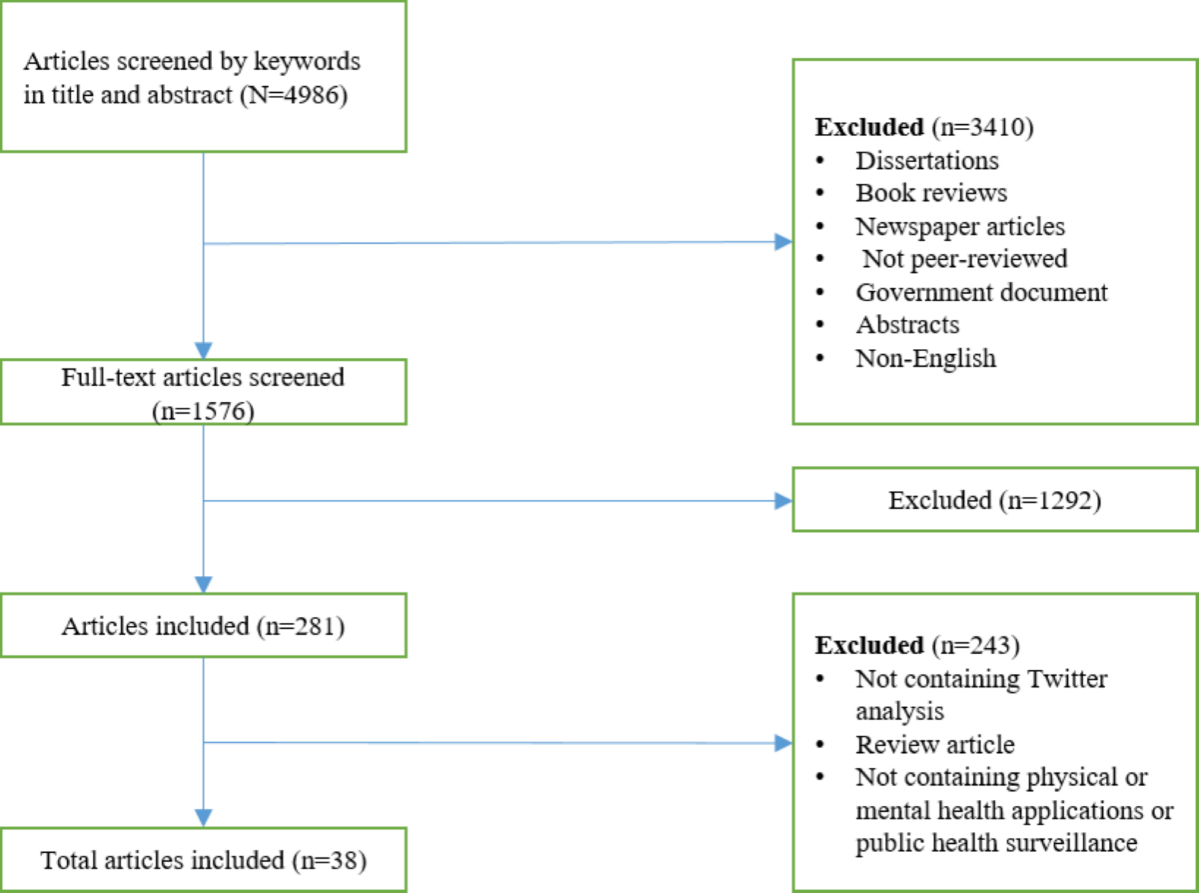
PRISMA (Preferred Reporting Items for Systematic Reviews and Meta-Analyses) procedural flowchart.

The search strategy successfully identified >1576 peer-reviewed articles. Of these, 38 (Table 1) met the eligibility criteria (see Figure 1 for the PRISMA flowchart). This section presents the literature that used Twitter as a data source for (1) language analysis to identify health threats and physical and mental health understandings about people and societies and (2) public health surveillance related to the leading causes of mortality representing 3 primary categories (respiratory infections: 10/38, 26%; cardiovascular diseases: 3/38, 8%; and COVID-19: 4/38, 11%). Articles included results on analyzing the contents of tweets to study influenza rates; heart disease; cardiac arrest; COVID-19; well-being; substance use; and mental health conditions such as attention-deficit/hyperactivity disorder (ADHD), posttraumatic stress disorder (PTSD), and depression.

**Table 1:** List of studies included in this scoping review grouped by public health issue (n=38).

Study, year		Sample	Methods	Results and conclusions	Themes
**Noninfectious diseases**					
	Paul and Dredze [22], 2011	Subset of 1.63 million health-related tweets depending on the experiment; May 2009 to October 2010	Modified the ATAM^a^ to incorporate previous knowledge. Annotated the 20 ailments with disease names. Compared the ailment distributions with distributions estimated from WebMD articles. Calculated correlations between each risk factor and the related ailments measured for each US state that had ≥500 tweets. Computed the normalized rate of the allergy ailment by state and by month.	ATAM+ outperformed ATAM, yielding a higher correlation with CDC^b^ influenza data. Treatments were more consistent than symptoms. Strongest correlation between risk factor and ailment: tobacco use and cancer ailment. Observed the same patterns of allergies stated in WebMD. Twitter contains many different types of information of value to public health research on many different ailments.	LA^c^
	Bosley et al [23], 2013	62,163 tweets about cardiac arrest; April 2011 to May 2011	Characterized tweets by content, dissemination, and temporal trends as well as authors’ self-identified background, tweet volume, and followers.	A total of 25% of the sample included resuscitation and cardiac arrest–specific information. Resuscitation-specific tweets were posted primarily on weekdays. Users with ≥16 resuscitation-specific tweets in the study time frame had a mean of 1787 followers and mostly self-identified as having a health care affiliation. Health care providers can distill tweets by user, content, temporal trends, and message dissemination to better engage via social media.	LA and PHS^d^
	Paul and Dredze [12], 2014	144 million health-related tweets; August 2011 to February 2013	Tested 2 models: LDA^e^ and the ATAM. Calculated the prevalence of ailments over time and geographic regions.	ATAM discovered more human-identifiable ailments with higher coherence than LDA. Discovered 13 clusters of tweets that correlated with temporal surveillance data and geographic survey data. With minimal human supervision and no historical data for training, a single general-purpose model can identify many different health topics in social media that correlate with ground truth data.	LA and PHS
	Eichstaedt et al [13], 2015	148 million county-mapped tweets across 1347 counties; June 2009 to March 2010	Characterized community-level psychological correlates of age-adjusted mortality from AHD^f^. Created a cross-sectional regression model based only on Twitter language to predict AHD mortality.	Discovered risk factors and protective factors. Quantified the correlation between risk and protective factors and AHD mortality. Predicted AHD mortality better than a model combining 10 common demographic, socioeconomic, and health risk factors. Capturing community psychological characteristics through social media is feasible, and these characteristics are strong markers of cardiovascular mortality at the community level.	LA and PHS
	Sinnenberg et al [4], 2016	10 billion tweets filtered for keywords about cardiovascular disease; random subset of 2500 tweets hand coded for tweet content and modifiers; July 2009 to February 2015	Characterized tweets relative to estimated user demographics.	Diabetes (n=239,989) and myocardial infarction (n=269,907) terms were used more frequently than heart failure terms (n=9414). Users tweeting about cardiovascular disease were more likely to be older and female. Most tweets (94%) were health related. Common themes: risk factors (42%), awareness (23%), and management (23%). Twitter shows promise to characterize public understanding and communication about heart disease.	LA and PHS
	Guntuku et al [6], 2017	1.3 million tweets by 1399 Twitter users with self-reported diagnoses of ADHD^g^	Comparing Twitter language with that used by a control set matched by age, gender, and period of activity	A review of linguistic themes in posts revealed notable distinctions in the way users described self-efficacy, emotional regulation, negation, self-criticism, substance use, and feelings of exhaustion. Individuals with ADHD tend to be less agreeable and more forthcoming in their posts and exhibit distinct posting habits in terms of frequency and timing compared with controls.	LA
**Infectious diseases**					
	Ritterman et al [24], 2009	48 million tweets; April 10 to June 11, 2009.	Performed regression using the SVM^h^ trained on all extracted features on the prices of the prediction market for all days except the current one. Calculated how far the model’s forecast deviated from the actual prediction market price.	Historical context from tweets improved forecast accuracy. Outperformed baseline methods based purely on market time-series information. Information in noisy social media (eg, Twitter) can be used as a proxy for public opinions.	LA and PHS
	Chew and Eysenbach [25], 2010	2 million tweets about swine influenza, May 2009 to December 2009; randomly selected 5395 tweets from 9 days, 4 weeks apart	Used the Infovigil infoveillance system to archive tweets and coded them using a triaxial coding scheme. Created database queries for keywords and correlated these results with manual coding. Tracked tweet content and tested the feasibility of automated coding.	“H1N1” use increased from 8.8% to 40.5%, indicating a gradual adoption of WHO^i^ terminology. Identified posts as resource-related (52.6%) and misinformation (4.5%). Most popular source: news websites (23.2%) versus government and health agencies (1.5%). Tweets correlated with H1N1 incidence. H1N1-related tweets were primarily used to disseminate information from credible sources but were also a source of opinions and experiences. Tweets can be used for real-time content analysis and knowledge translation research.	LA and PHS
	Culotta [26], 2010	574,643 public tweets containing common words (“a,” “I,” “is,” “my,” “the,” “to,” and “you”); February 2010 to April 2010	Developed 10 regression models that predict ILI^j^ rates based on the frequency of tweets containing keywords.	Simple bag-of-words classifier trained on roughly 200 documents effectively filtered erroneous document matches, resulting in better model predictions (0.78 correlation with CDC data).	LA and PHS
	de Quincey and Kostkova [27], 2010	135,438 “flu”-containing tweets from 70,756 users; May 7 to 14, 2009	Collected and counted the most recent tweets each minute containing “flu” for 1 week using the Twitter API^k^	Frequency of influenza-related words (eg, “flu”: n=138,260; “Swine”: n=99,179) and preliminary collocation of words next to “flu” (eg, “Swine” 1 word to the left: n=96,651; “Cases” 1 word to the right: n=6194). Highlighted the potential for Twitter to be used in conjunction with preexisting EI^l^ tools. Real-time Twitter reports about users’ own illnesses, illnesses of others, or confirmed cases from the media are both rich and highly accessible.	PHS
	Lampos and Cristianiani [28], 2010	Average of 160,000 daily geotagged tweets over 24 weeks	Searched for symptom-related statements and turned statistical information into an influenza score.	Linear correlation of >95% between influenza score and official data. Tweets contain data completely independent of data commonly used for these purposes, can be used at close time intervals, and constitute an early warning in various situations but mostly can give timely and free information to health agencies to plan health care.	PHS
	Aramaki et al [29], 2011	400,000 “influenza”-containing tweets; November 2008 to June 2010.	Annotated a set of tweets as positive or negative—that is, whether they were both (1) about a person or surrounding persons with influenza and (2) in the affirmative present or recent past tense. Built a tweet classifier and compared detection performance with official reports.	SVM-based classifier achieved the highest performance (0.890 correlation) when news was not excessive but suffered from an avalanche of news, generating a news bias. This correlation is considerably higher than the query-based approach, demonstrating the basic feasibility of the proposed approach. Twitter texts reflect the real world, and NLP^m^ techniques can be applied to extract only tweets that contain useful information.	PHS
	Diaz-Aviles and Stewart [30], 2011	456,226 tweets about the German EHEC^n^ outbreak; May 2011 to June 2011.	Tracked EHEC tweets and ground truth data on cases. Computed a low-dimensional representation of the data using hashtagging behavior on Twitter and the topic result of applying LDA. Conducted a user study with experts to determine if reranking strategies should be based on LDA topics or hashtags.	A total of 9 early tweets were enough to generate an alarm on May 20, 2011, a day ahead of well-established early warning systems. Both reranking methods discovered new relationships that helped identify more relevant tweets. LDA-boosted ranking performance was more expensive than tracking recurring hashtags. Twitter can be exploited to support EI in the tasks of early warning, signal assessment, and outbreak investigation.	PHS
	Gomide et al [31], 2011	Data set one: 12,256 dengue-related geotagged tweets, January 2009-May 2009; data set two: 465,444 dengue-related geotagged tweets, December 2010 to April 2011	Speculated how users refer to dengue in tweets using sentiment analysis and focused only on tweets that expressed personal experience with dengue. Constructed a linear regression model to predict dengue incidence. Created an active surveillance methodology based on 4 dimensions: volume, location, time, and public perception.	Correlation of 0.9578 between official cases and tweets posted during the same period. Quality of spatiotemporal clusters comparable with official data. Twitter can be used to spatiotemporally predict dengue epidemics via clustering.	PHS
	Signorini et al [32], 2011	Data set one: 951,697 influenza-related tweets, April 29 to June 1, 2009; data set two: 4,199,166 influenza-related tweets, October 2009 to December 2009	Tracked rapidly evolving public sentiment and actual disease activity for H1N1 or swine influenza.	Tweets not only tracked users’ interest and concerns related to H1N1 influenza but also estimated disease activity in real time, that is, 1-2 weeks faster than current practice allows. Twitter can be used as a measure of public interest or concern about health-related events.	PHS
	Achrekar et al [33], 2012	4.5 million influenza-related tweets; 2009 to 2010.	Implemented an ARX^o^ model using current Twitter data and CDC ILI rates from previous weeks to predict current influenza statistics.	Twitter data correlated with ILI rates across various US regions and effectively improved prediction accuracy. For most of the regions, Twitter data best fit age groups of 5-24 and 25-49 years (likely the most active user age groups). Although previous ILI data from the CDC offer a true (but delayed) assessment of an influenza epidemic, Twitter data provide a real-time assessment of the current epidemic condition and can be used to compensate for the lack of current ILI data.	PHS
	Chunara et al [34], 2012	Data set one: 65,728 cholera-related tweets during the initial outbreak, October 20 to November 3, 2010; data set two: 84,992 cholera-related tweets during Hurricane Tomas, November 3 to December 1, 2010. Included tweets containing “cholera” in English or French.	Assessed the correlation among the volume of cholera-related HealthMap news media reports, tweets, and government cholera cases reported in the first 100 days of the 2010 Haitian cholera outbreak.	Informal source volume correlated with official case data and was available up to 2 weeks earlier. Reproductive number estimates were similar using HealthMap or Twitter or official data during the initial outbreak and when Hurricane Tomas afflicted Haiti. At the early stages of an outbreak, informal sources can be indicative of the fact that an outbreak is occurring and also highlight disease dynamics by estimating the reproductive number.	PHS
	Sadilek et al [35], 2012	6237 users with ≥101 tweets each geolocated in NYC^p^ (2,535,706 total tweets)	Constructed a probabilistic model that predicted if and when an individual fell ill with high precision and good recall using Twitter-based social ties and colocations with other people.	Predicted with 90% confidence (1) a total of 10% of cases a week before they occurred and (2) almost 20% of cases a day in advance. The health of a person can be accurately inferred from location and social interactions observed via social media. Future health states can be predicted with consistently high accuracy more than a week into the future.	PHS
	Szomszor et al [36], 2012	2,993,022 “flu”-containing tweets; May 2009 to December 2009	Calculated the normalized cross-correlation ratio between various signals from Twitter and official surveillance data.	Strong correlation of official data with self-reporting tweets but not with the signals for all influenza tweets, those containing links, and retweets. Twitter detected infection-spreading events up to 1 week before conventional surveillance data. Twitter can serve as a self-reporting tool and, hence, provide indications of increased infection spreading.	PHS
	Park et al [37], 2020	43,832 Twitter users and 78,233 relationships derived from approximately 18,000 tweets containing COVID-19–related terms (in Korean)	Generated 4 networks in terms of key issues regarding COVID-19 in South Korea. Compared how COVID-19–related issues circulated on Twitter through network analysis. Classified top news channels shared via tweets. Conducted a content analysis of news frames used in the top-shared sources.	Faster spread of information in the coronavirus network than in others (Corona19, Shincheon, and Daegu). People who used the word “Coronavirus” communicated more frequently with each other, spread information more quickly, and exhibited a lower diameter than those who used other terms. Most of the popular news on Twitter had nonmedical frames, but the spillover effect of news articles that delivered medical information about COVID-19 was greater. Monitoring public conversations and media news that propagates rapidly can assist public health professionals in their complex and fast-paced decision-making processes.	PHS
	Singh et al [38], 2020	2,792,513 tweets with COVID-19–related hashtags, including geotagged tweets; January 2020 to March 2020	Identified common themes in tweets about COVID-19 and how the prevalence of these themes changed over time. Searched for 10 common myths in the Twitter corpus. Quantified high- or low-quality health sources and credible media sources shared on Twitter.	Conversations about COVID-19 continued to grow. Tweets correlated with COVID-19 cases and led cases by 2-5 days. Predominant themes: health or the virus itself or the global nature of the pandemic. Misinformation and myths were relatively low in volume. Credible health sources were in original tweets as often as but were retweeted less often than unreliable sources. Tweets can help predict the spread and outbreak of COVID-19 when other reliable leading indicators are unavailable.	PHS
	Pobiruchin et al [39], 2020	21,755,802 COVID-19–related tweets; February 2020 to April 2020	Filtered tweets based on 16 COVID-19–related hashtags and extracted each tweet’s text, corresponding metadata, and user profile. Applied a link categorization scheme to the top 250 shared resources and analyzed the relative proportion for each category. Analyzed temporal variations of global tweet volumes specifically for the European region.	COVID-19–related tweets increased after the WHO announced its name on February 11, 2020, and stabilized in late March at a high level. Most shared resources were from social media platforms. The most prevalent category in the top 50 shared resources was “Mainstream or Local News.” For the category “Government or Public Health,” only 2 information sources were found in the top 50: the CDC (rank 25) and the WHO (rank 27). The naming of the disease by the WHO was a major signal to address the public audience with a public health response via social media platforms such as Twitter. It is important to monitor the spread of fake news during a pandemic.	PHS
	Cuomo et al [40], 2021	59,937 COVID-19–related tweets geolocated to US counties; March 2020	Leveraged an SVM classifier to obtain a larger set of geocoded tweets with characteristics of users self-reporting COVID-19 symptoms, concerns, and experiences. Assessed the longitudinal relationship between identified tweets and the number of officially reported COVID-19 cases using linear and exponential regression at the US county level. Analyzed changes in tweets that included geospatial clustering for the top 5 most populous US cities.	Tweet volume increased during the study period with variation between city centers and residential areas. Tweets identified as reporting COVID-19 symptoms or concerns were more predictive of active cases as temporal distance increased. Social media communication dynamics during the early stages of a global pandemic may exhibit geospatial-specific variations among different communities.	PHS
**Mental health and well-being**					
	de Choudhury et al [41], 2013	69,514 tweets: 23,984 depression-indicative tweets and 45,530 standard posts	Developed a probabilistic model trained on a corpus of tweets and users’ social activity, emotion, and language.	Predicted depression-indicative tweets with 73% accuracy. Introduced a social media depression index that closely mirrors CDC data. Demonstrated the potential of using social media as a reliable tool for measuring population-scale depression patterns.	LA
	Seabrook et al [42], 2018	Status updates and depression severity ratings of 29 Facebook and 49 Twitter users collected through the MoodPrism app	Computed the average proportion of positive and negative emotion words used, within-person variability, and instability.	Negative emotion word instability was a significant (*P*=.02) predictor of greater depression severity on Facebook, but the opposite pattern emerged on Twitter. Negative emotion word instability may be a simple yet sensitive measure of time-structured variability, but its usefulness may depend on the social media platform.	LA
	Jaidka et al [43], 2020	1.53 billion geotagged tweets in English	Systematically evaluated various techniques for analyzing text, including word-level and data-driven methods, to generate well-being estimates for 1208 counties in the United States, followed by comparing the estimates from Twitter data with those from the Gallup-Sharecare Well-Being Index survey, which was based on 1.73 million phone surveys, to evaluate the effectiveness of the different text analysis methods in providing accurate well-being estimates at the county level.	Using word-level methods such as LIWC^q^ 2015 and Language Assessment by Mechanical Turk (labMT) resulted in inconsistent well-being measurements at the county level because of variations in language use caused by regional, cultural, and socioeconomic factors. However, by eliminating just a small number of the most commonly used words, significant improvements in the accuracy of well-being predictions were observed. Regional well-being estimation from social media data may be robust when supervised data-driven methods are used.	LA
**Substance use**					
	West et al [44], 2012	5,697,008 tweets from Twitter users in 9 states: 4,727,046 tweets from October 2010 and 969,962 tweets from New Year’s Eve 2010	Identified tweets that contained words reflective of problem drinking from a list of slang words (not mere mentions of alcohol). Compared spatiotemporal data between tweets in 2 periods.	Twitter users were most likely to tweet about problem drinking on Friday, Saturday, and Sunday during the hours of 10 PM to 2 AM. Tweets during the New Year’s Eve holiday were twice as common as tweets on weekends in October. Tweets about problem drinking corresponded with expected periods of actual problem drinking. Social norm interventions may be an effective tool in correcting misperceptions related to problem drinking by informing Twitter followers that problem drinking behaviors are not normative.	LA and PHS
	Myslin et al [45], 2013	7362 tobacco-related tweets at 15-day intervals; December 2011 to July 2012	Manually classified tweets using a triaxial scheme (genre, theme, and sentiment). Used naive Bayes, k-nearest neighbor, and SVM algorithms to train machine learning classifiers that detect tobacco-related versus irrelevant tweets and positive versus negative sentiment. Computed φ contingency coefficients between each of the categories to discover emergent patterns.	Most prevalent genres: first- or secondhand experience and opinion. Most frequent themes: hookah, cessation, and pleasure. Tobacco sentiment was more positive than negative. Hookah and e-cigarettes had a positive sentiment, while traditional tobacco products and general references had a negative sentiment. SVMs using a relatively small number of unigram features (500) achieved the best performance in discriminating tobacco-related from unrelated tweets. Machine classification of tobacco-related posts shows a promising edge over strictly keyword-based approaches, yielding an improved signal to noise ratio in Twitter data and paving the way for automated tobacco surveillance applications.	LA and PHS
	Kershaw et al [46], 2014	31.6 million geotagged tweets; November 2013 to January 2014	Grouped tweets by location in a given hour and detected alcohol-related terms to create a SMAI^r^. Compared SMAI with ground truth data.	Correlation between SMAI and ground truth at national and local levels with 97% accuracy. Detected both shifts away from typical alcohol consumption (holidays and celebrations) and lags in terms such as “hangover” spiking 12 to 24 hours after spikes in “drunk.” Twitter could be used to assess policing levels in town centers and staffing levels in emergency departments.	PHS
	Daniulaityte et al [47], 2015	27,018 geotagged dabs-related tweets out of a general sample of 209,837 tweets; October 2014 to December 2014	Calculated percentages of dabs-related tweets per state adjusted for different levels of overall tweeting activity for each state. Compared adjusted percentages of dabs-related tweets among US states with different cannabis legalization policies.	Adjusted percentages of dabs-related tweets were highest in states that legalized recreational or medicinal cannabis use.	PHS
	Thompson et al [48], 2015	1% random sample of 7,290,100 marijuana-related tweets posted in the second weeks of March 2012 to May 2012 and May 2013 to July 2013. Preprocessed to 36,939 original and approximately 10,000 reposted tweets.	Categorized tweet content (eg, mention of personal marijuana use, parents’ views, and perceived effects). Extracted self-reported age from available tweet metadata. Compared self-identified adolescents versus others and pre- versus postelection content.	Most (n=1928, 65.6%) original tweets by adolescents reflected a positive attitude toward marijuana, and 42.9% indicated personal use. A total of 36% of adolescents’ tweets that mentioned parents indicated parental support for the adolescents’ marijuana use. Tweet volume about personal marijuana use and positive perceptions of it increased. Adolescents and others on Twitter are exposed to positive discussion normalizing use.	PHS
	Das and Kim [49], 2015	3416 tweets geotagged to the Bay Area manually verified to contain alcohol-related terms; June 2013 to July 2013	Generated an “epidemic curve” by plotting the frequency of tweets each day during the data collection period. Used Esri ArcGIS to plot each tweet’s GPS coordinates and create heat maps.	Alcohol tweet volume followed a consistent, cyclical pattern, with tweets slowly rising as the weekend approached and declining on Monday. Alcohol tweet volume was higher during festivals and national holidays than on nonholiday weekends. Tweets were clustered in the Market Street corridor and in the Castro district—the location of many bars and Gay Pride–associated festivities. Through machine learning strategies that can automatically review tweets, public health departments could rapidly create heat maps of alcohol-related tweets, discover alcohol hot spots hidden from epidemiological surveys, and then tailor interventions to these high-risk areas.	PHS
	Cabrera-Nguyen et al [50], 2016	587 Twitter users aged 18 to 25 years; February 2014	Shared a self-administered web-based survey that assessed current heavy episodic drinking and current marijuana use, exposure to proalcohol and promarijuana content on Twitter, and demographic covariates.	Current heavy episodic drinking was significantly associated with higher levels of exposure to proalcohol content. Current marijuana use was significantly associated with higher levels of exposure to promarijuana content. In-depth research regarding young adults’ exposure to proalcohol- and promarijuana-related content via Twitter may provide a foundation for developing effective prevention messages on this social media platform to counter the proalcohol and promarijuana messages.	PHS
	Baumgartner and Peiper [51], 2017	2,199,042 Twitter users comprising a 2-hop network seeded from cannabis dispensary Twitter accounts	Extended stochastic block modeling to empirically derive communities of cannabis consumers as part of a complex social network on Twitter.	Found candidate samples of medical, recreational, and illicit cannabis users. Most frequent codes: promotion, lifestyle, business, recreational, and professional. Correlations: spam—international (0.81), dispensary—collective (0.45), recreational—promotion (−0.28), professional—advocacy (0.29), business—marketing (0.26), and entertainment—meme (0.26). The creation of state repositories of dispensary accounts will serve as the basis for monitoring cannabis consumers while incorporating internet-related measures into intervention design and outcome evaluation.	PHS
	Curtis et al [52], 2018	138 million county-mapped tweets; October 2011 to December 2013. Further filtered to those with corresponding EAC^s^ rates, county-level SES^t^ and demographic variables, and ≥40,000 tweeted words from a random 1% sample.	Used predictive modeling, differential LA, and mediating LA.	Tweets accurately predicted EAC rates, and Twitter topics explained much of the variance between socioeconomic variables and EAC. Twitter data can be used to predict public health concerns such as EAC. Using mediation analysis in conjunction with predictive modeling allows for a high portion of the variance associated with SES to be explained.	PHS
	Anwar et al [53], 2020	Random sample of 10,000 tweets from 100,777 opioid-related tweets geotagged to North Carolina; January 2009 to December 2018	Identified opioid-related terms by analyzing word frequency for each year and compared patterns of opioid-related posts with official OOD^u^ data.	Tweet patterns about prescription opioids, heroin, and synthetic opioids resembled the triphasic nature of OODs. Tweet counts were unrelated to prescription OODs but were associated with heroin and synthetic OODs in the same year and the following year. Heroin tweets in a given year predicted heroin deaths better than lagged heroin OODs alone. Findings support using Twitter data as a timely indicator of opioid overdose mortality, especially for heroin.	PHS
	Allem et al [54], 2020	60,861 cannabis-related tweets; May 2019 to December 2019.	Distinguished between posts from social bots and nonbots. Used text classifiers to identify topics in posts.	Topics: using cannabis with mentions of cannabis initiation; processed cannabis products; and health and medical with post suggesting that cannabis could help with cancer, sleep, pain, anxiety, depression, trauma, and PTSD^v^. Polysubstance use with cannabis: cocaine, heroin, ecstasy, LSD^w^, methamphetamines, mushrooms, and Xanax. Social bots regularly made health claims about cannabis. Processed cannabis products, unsubstantiated health claims about cannabis products, and the co-use of cannabis with legal and illicit substances warrant consideration by public health researchers in the future.	PHS
	Giorgi et al [55], 2020	19.3 million “drunk” tweets, 3.3 million geolocated and filtered to counties with ≥1000 words within the drunk tweets: random 10% sample, June 2009 to April 2014+random 1% sample, April 2014 to February 2015	Selected tweets containing “drunk” and clustered the words and phrases distinctive of drinking posts into topics and semantically related sets. Correlated geolocated “drunk” tweets with the prevalence of self-reported EAC. Identified linguistic markers associated with excessive drinking in different regions and cultural communities.	EAC correlated with “Drunk” tweet volume at county and state levels as well as references to drinking with friends and family and driving under the influence. Cultural markers of drinking: religious communities had a high frequency of anti–drunk driving tweets, Hispanic centers discussed family members drinking, and college towns discussed sexual behavior. Twitter can be used to explore the specific sociocultural contexts in which excessive alcohol use occurs within particular regions and communities.	LA and PHS

^a^ATAM: Ailment Topic Aspect Model.

^b^CDC: Centers for Disease Control and Prevention.

^c^LA: language analysis.

^d^PHS: public health surveillance.

^e^LDA: latent Dirichlet allocation.

^f^AHD: atherosclerotic heart disease.

^g^ADHD: attention-deficit/hyperactivity disorder.

^h^SVM: support vector machine.

^i^WHO: World Health Organization.

^j^ILI: influenza-like illness.

^k^API: application programming interface.

^l^EI: epidemic intelligence.

^m^NLP: natural language processing.

^n^EHEC: enterohemorrhagic *Escherichia coli*.

^o^ARX: autoregression with exogenous input.

^p^NYC: New York City.

^q^LIWC: Linguistic Inquiry and Word Count.

^r^SMAI: Social Media Alcohol Index.

^s^EAC: excess alcohol consumption.

^t^SES: socioeconomic status.

^u^OOD: opioid overdose deaths.

^v^PTSD: posttraumatic stress disorder.

^w^LSD: lysergic acid diethylamide.

### Twitter for Public Health Surveillance: Leading Causes of Mortality

#### Overview

Twitter analysis has the potential to be precise, cost-effective, and practical for keeping track of people’s behaviors, health outcomes, emergencies, and disease outbreaks. The process of public health surveillance systematically collects and analyzes health-related data for disseminating information about public health practices and evaluating implemented methods [56]. Surveillance offers early warnings for approaching calamity, records for monitoring and evaluation, and evidence of disease transmission [56]. Although conventional clinical databases were previously the main available surveillance frameworks, digital technology innovations have changed the conduct of public health surveillance [57]. Researchers have found that digital surveillance methods provide advantages (eg, timeliness and cost) over the more conventional public health surveillance systems [58]. They supplanted passive surveillance methods (eg, physician case reports to local health departments) and active methods (eg, local health department activity searches for other cases) [58]. In this section, we discuss the literature that demonstrates how Twitter data were used for real-time surveilling of chronic illnesses, disease epidemics, and problematic behaviors to support and improve public health.

#### Influenza Surveillance

Influenza was the first and most common disease to be examined using Twitter. Several exploratory studies (9/38, 24%) on influenza targeting Twitter users from the United States and the United Kingdom between 2009 and 2012 used primary keyword searches from “influenza” or “flu” [27-29,36] to more definite influenza subtypes such as H1N125 [24,25,29] and symptomatic influenza groups [26,32,35]. A study by Signorini et al [32] searched important terms such as “influenza,” “infection,” and “hospital” to track Twitter users’ concerns during the 2009 H1N1 pandemic. A similar study by Sadilek et al [35] identified the health of Twitter users through geotagging (ie, geographical identification) to potentially predict influenza transmission. Twitter was shown to predict influenza epidemics 2 weeks before traditional surveillance methods used by the Centers for Disease Control and Prevention [32].

#### Escherichia Coli and Dengue Fever Surveillance

Recent studies used Twitter data to conclude whether a rapid opportunity for the detection of further infectious disease occurrences, such as *E coli*, cholera, and dengue fever, can be improved. A study by Diaz-Aviles and Stewart [30] analyzed >6 million Twitter posts on health conditions during the 2011 *E coli* outbreak in Germany. The authors identified almost 500,000 tweets related to *E coli* and determined that users’ tweets would have detected the epidemic 24 hours earlier than traditional warning surveillance methods. In a study by Gomide et al [31], the authors proposed a dengue fever surveillance system that demonstrated a correlation between the number of dengue cases reported by Brazil’s official statistics and tweets based on the volume, location, time, and public perception of users. In a similar study, Chunara et al [34] found that cholera-related tweets could anticipate earlier estimations of news trends and reported cases 2 weeks earlier than Haiti health authorities reported during the first 3 months of the 2010 Haitian cholera outbreak. Surveillance of disease outbreaks using Twitter data as a tracking system appeared to have timeliness advantages in numerous infectious disease epidemic settings [20].

#### COVID-19 Surveillance

Twitter activity increased substantially during the COVID-19 pandemic. Recent surveillance studies worldwide used these data to assess the unprecedented impact caused by the disease. The studies examined the rapid dissemination of COVID-19 information and misinformation and news-sharing behaviors [37-39] and identified high Twitter volume as a potential predictor for confirmed cases [38,40]. Specifically, increased volumes of COVID-19–related tweets were observed in large European cities with more concentrated populations [39] following the World Health Organization announcement releasing the name of the nascent disease. In the United States, high volumes of COVID-19–related tweets were analyzed to monitor the trends of information spread and were linked to longitudinal trends in local infection rates [40]. For example, highly concentrated cities such as New York City contained areas such as Manhattan with very high volumes of tweets linked to extremely high infection rates in the early stages of the pandemic [40]. Similarly, but from a global perspective, Singh et al [38] found that confirmed COVID-19 cases were highly associated with the location of COVID-19–related tweets. Their results indicated that users sharing COVID-19–related tweets were mainly from China, followed by other epicenter countries such as the United States, India, Iran, and Italy. The studies also shed light on news-sharing behaviors and the fast dissemination of news information and misinformation via Twitter. South Korean users tweeted or retweeted medical-related news information more often and quickly than nonmedical information, and users who included the word “Coronavirus” were more likely to engage with each other more frequently [37]. COVID-19–related information was widely tweeted, but research also found that users shared a substantial amount of misinformation that might have driven confusion and mistrust in governmental initiatives to combat the pandemic. Although a high volume of incredible information was shared, Singh et al [38] reported that credible information was lateral to incredible information. Overall, the results from these studies suggested that COVID-19–related tweets may help understand the impact of COVID-19 on news-sharing behaviors, public responses, and predictions of potential infectious outbreaks and reproduction when other reliable surveillance measurements are not readily available.

#### Cardiovascular Disease Surveillance

Of the 38 studies, 3 (8%) exploratory studies targeting cardiovascular disease focused on analyzing Twitter data to determine mortality rates and the public’s understanding and communication about cardiovascular disease. As stated previously, Twitter linguistics were used to predict heart disease mortality rates based on community-level language patterns reflecting negative emotions that emerged as risk factors and positive emotions that emerged as protective factors [13]. Twitter posts that contained text related to heart disease included information about risk factors, management, and awareness that could enhance cardiovascular disease surveillance and market to precise users in need of medical assistance [4,23]. Sinnenberg et al [4] examined 2500 heart-related tweets and topics, successfully tracked health status in real time, and characterized the public understanding and communication about heart disease. In summary, the studies showed that Twitter data could be a valuable tool for surveilling mortality rates and monitoring real-time changes in discussions about heart disease. In addition to using Twitter data as a surveillance tool for cardiovascular disease, many scholars found effective ways to monitor substance use behaviors.

#### Substance Use Surveillance

Twitter has been a promising platform for surveying substance use such as alcohol, tobacco, cannabis, and other drugs as Twitter users often publicly share and display their substance use activities via substance use–related content. The studies used Twitter to examine emerging trends in alcohol [44,52,55] and cannabis use [47,48,51,54], track alcohol [46,49,52] and opioid abuse [53] using geocoded data, study the effect of proalcohol and procannabis content on use among young adults [50], and identify the beliefs and behaviors of youth alcohol and tobacco use patterns [45]. Traditional surveillance methods could not provide real-time, population-based surveillance for substance use, but mining tweets provided real-time surveillance that served as a population-based approach to investigating substance use trends and trajectories [49].

#### Summary

In this section, studies leveraging Twitter data centered on understanding how diseases are dispersed through a population and predicting emerging public health trends by tracking infection rates, health status (eg, cardiovascular disease), and consumption (eg, substance use) in real time. The studies looked beyond a better insight into disease spread and health trends. However, they also gained deep insights into monitoring the public health state of disease occurrence, symptoms, and outbreaks while characterizing the public understanding and communication about a disease. In summary, Twitter data can benefit public health actions in disease and behavioral surveillance, showing positive results at the individual and community levels. In the following section, we will review the literature that used language analyses and approaches to process and analyze Twitter data to identify and understand physical and mental health conditions as mental health problems can affect physical health conditions and are commonly associated with the risk of chronic diseases and substance use.

### Language Analysis to Study Physical and Mental Health

Before exploring research on the relationship between physical health, mental health, and Twitter language, it is vital to understand how the linguistic analysis of large data sets is carried out. A variety of language analyses were used descriptively to identify and monitor emerging health threats (eg, influenza outbreaks) [26]. Moreover, these analyses provided health and psychological insights into people and societies to make predictions about a variety of health-related outcomes [1]. To conduct these analyses, researchers used an automatic computer process to extract the relative frequencies of single words, phrases (2 or 3 consecutive words), and topics across millions of users’ tweets [6,13]. Of the 38 articles included in this review, 8 (21%) analyzed tweets’ language about a specific health topic to characterize public discourse on Twitter. Within this group, 1 subcategory focused on sentiment analysis—the process of deriving opinions, feelings, and subjectivity in texts [59], such as whether users’ tweets convey positive, negative, or neutral sentiments [60].

The tremendous amount of personal information shared on Twitter can be examined to provide physical health data [15]. For example, Bosley et al [23] and Eichstaedt et al [13] used Twitter to gain insights into cardiovascular disease based on users’ language. Both characterized >112,000 tweets about cardiovascular issues and resuscitation by identifying keywords such as “cardiac arrest,” “heart disease,” and “cardiopulmonary resuscitation.” The tweets were characterized by content, dissemination, and temporal trends (days and times with a high volume of tweets on a topic). By examining 50,000 tweets, Eichstaedt et al [13] used a cross-sectional regression model based on Twitter language to predict mortality rates from atherosclerotic heart disease. Bosley et al [23] identified 62,163 tweets using 7 search keywords in a 38-day time frame. They found that 25% of users’ tweets contained resuscitation and cardiac arrest information. Twitter users shared linguistic information related to cardiac arrest and resuscitation, including risk factors, symptoms, training, education, screenings, and other information. The findings of these 2 studies indicated that Twitter language data can be mined to detect death rates, identify heart-related content, and better understand how heart health–related information is shared and discussed.

The language analysis of tweets may also be beneficial for understanding other health-related conditions. Influenza was the most frequently analyzed disease using Twitter data [12] by evaluating and characterizing the language used. Twitter users frequently freely tweeted private content; tweets similar to “sick with the flu” and “I got the flu” are typical posts [22]. Although realizing that 1 user has the seasonal influenza virus might not be particularly intriguing, millions of such tweets can be revealing, for instance, to follow the influenza rate in the United States [26]. For example, Culotta [26] mined Twitter discussions as a way to validate Twitter’s role as a method of warning practitioners and health officials about influenza epidemics in the United States. More recent influenza epidemics such as H1N1 were successfully analyzed to evaluate the general public’s awareness of public health advice [25]. In 3% (1/38) of the studies, researchers analyzed 2 million tweets containing the key terms “swine flu” and “H1N1” using Infovigil, an “infoveillance” system, and found elevated levels of “precise knowledge among the public” [25]. The study recommended that Twitter mining may be an innovative method for health officials to “measure public awareness of their campaigns and respond to shifting concerns in real time” [25]. Another influenza study by Ritterman et al [24] stated that analysis of tweets about influenza-related keywords could enhance prediction model precision by giving advance notices of severe occurrences, for example, the H1N1 epidemic.

Analyzing Twitter messages can also have a more substantial impact on public health informatics than monitoring influenza rates [22]. A recent study by Guntuku et al [6] focused on adult behaviors with self-reported diagnoses of ADHD as described by users in their Twitter posts. The authors computationally analyzed 1.3 million tweets from nearly 1400 users to gain insights into the type of discussions and information shared and “how their language is correlated with users’ characteristics, personality, and temporal orientation” [6]. By analyzing users’ tweets, they found that Twitter users with ADHD were more active on the platform, less friendly, and posted more profanity. According to the researchers, the posting behaviors of Twitter users with self-proclaimed ADHD were, in fact, consistent with the symptoms of the disorder.

In addition to providing data for physical health conditions, researchers analyzed Twitter language to predict and determine emotional well-being [24,43] as well as various mental illnesses [10,41]. Psychological conditions, particularly depression [42] and PTSD [10], constitute a growing problem that affects millions of Americans. Unlike other health research using social media that relies on exact words or phrases related to a disease or health concern, research on mental illness has leveraged variations in language to suggest behavior changes. For example, a change in word use or frequency of posts signaled changes in a person’s mental health [10]. Similar to how other researchers extracted tweets to study influenza rates and predict heart disease, researchers used the same automatic computer process to extract the relative frequencies of words and phrases related to mental illness. However, another innovative process called crowdsourcing collected data by soliciting contributions from a large group of people, known as crowd workers, to examine mental health disorders on Twitter [41].

Twitter language is a potential source for measuring and predicting mental health conditions in people who use the platform. de Choudhury et al [41] identified users with major depression using crowdsourcing data on the presence of depression. They found that users with depression exhibited lower social media activity, more negative emotions, higher self-attentional focus, and more expressions related to religion. Similarly, Coppersmith et al [10] demonstrated that some Twitter users with PTSD were not difficult to identify when mining for Twitter posts that communicated definitive diagnoses rather than relying on conventional PTSD measurements. Coppersmith et al [10] used positive and negative PTSD language sentiments to train classifiers, which are language models that analyze probabilities that the same underlying process generates words or a thread of characters as the training data. The words were then divided into positive and negative classes. Using this technique, Coppersmith et al [10] captured a fundamental difference between Twitter users with and without PTSD from their Twitter language. The results revealed that users with PTSD posted substantially more Twitter messages using more negative emotions, anger, anxiety, and death-related words. On the basis of these 2 studies, analyses of users’ language on Twitter were deemed useful for detecting and identifying mental health conditions in individuals [41].

All the studies mentioned in this section analyzed the content of tweets and constructed various statistical language models (eg, PTSD classifiers). Statistical language models are algorithms for learning and capturing the critical statistical characteristics of the distribution of sequences of words in a language. These models use predictors representing expressions of positive and negative sentiment in Twitter language variables [61]. Many studies (18/38, 47%) also constructed predictive models using data mining and probability [62]. In addition, in recent years, deep learning and natural language processing have been widely used and are popular techniques embedded in many analytical models using Twitter data to study public health research. Leveraging these techniques and strategies, researchers used Twitter language for many functional tasks, ranging from providing insights into individual characteristics and personalities to continually monitoring positive and negative sentiments and predicting various health-related outcomes. In summary, the analysis of language on Twitter was practically unlimited. Individuals left digital footprints everywhere, expressing their emotions, behaviors, identities, personalities, and experiences [13].

### Limitations of the Existing Studies

A few limitations should be noted about the existing studies. A limitation of many Twitter-based studies was that Twitter users are only a subset of the general population. The Pew Internet and American Life Project [63] found that 31% of Twitter data tend to come from users aged between 18 and 29 years, in contrast to 19% of Twitter users being aged between 30 and 49 years, 9% of users being aged between 50 and 64 years, and <5% of users being aged >65 years. Therefore, there is a requirement for higher granularity in Twitter-based studies, especially for studies that leverage Twitter as a surveillance tool. Twitter analysis as a public health instrument raises ethical concerns as well. Regarding users’ privacy, some apprehensions include the use of the general population’s information without verbal or written consent as well as individuals lacking informed expectations regarding how their data are being used as part of public health research [64]. Twitter has the potential to help facilitate information sharing but might also be problematic in disseminating false information during standard and seasonal health events and crises. The literature also provides limited insights into how to decrease the greater possibilities of inaccurate information being mistakenly or deliberately shared with individuals searching for health information on the internet, creating accountability and authenticity challenges [65].

## Discussion

### Principal Findings

This scoping review provided a comprehensive synthesis of current research, leveraging Twitter data to analyze users’ generated language to explore and understand physical and mental health conditions and remotely monitor the leading causes of mortality related to emerging disease epidemics and risk behaviors. From the 38 reviewed empirical articles that were published within the past 13 years using Twitter as a data source in public health, two themes were identified: (1) analyzing Twitter language to determine physical and mental health outcomes and (2) using Twitter for public health detection and surveillance. On the basis of the research reviewed in this study, we discovered that Twitter holds several distinct types of valuable evidence for the field of public health in several different conditions. Analyzing language on Twitter can serve as an innovative method to examine health-related conditions such as chronic illnesses, especially for mental illnesses such as depression [41] and PTSD [10]. We identified studies using Twitter data to analyze mental illness, with findings suggesting that people are more open to addressing their mental health issues on social media [41]. Finally, we observed trends in Twitter-based studies proving that analyzing millions of tweets can offer a beneficial understanding of population health, disease tracking, and surveillance and produce rich data that connect with public health metrics and knowledge [22].

We found that several published studies used a variety of sophisticated language analysis techniques and strategies to mine user-generated tweets to help detect a wide range of conditions such as mental illnesses (depression and PTSD) and ADHD symptoms, monitor emerging disease outbreaks, and provide a deeper insights into people’s personalities and behavioral characteristics at a large scale. Furthermore, similar to previous review studies [17,18,20], our findings suggest that, by users leaving digital footprints, studies could analyze Twitter language in a real-time and less time-consuming manner while also opening an unobtrusive window into understanding complex health and behavior conditions. In addition, our findings demonstrated that most surveillance studies (17/38, 45%) primarily focused on infectious diseases, partly because of their global importance. Notably, the studies focused excessively on the observation and surveillance of influenza rates. However, we identified studies with a scope to explore the utility of Twitter data to survey other infectious diseases such as COVID-19 and noninfectious diseases such as cardiovascular disease and substance abuse. Despite our efforts to report on these studies, these areas remain understudied, yet these diseases often represent an enormous health burden among general populations worldwide. Overall, our findings suggest that using language analytical techniques and surveillance via Twitter could have a long-lasting impact on the domain of mental and physical illnesses and health-related and disease surveillance, conveying the larger media lens to the public health field [20]. This effect was imperative in public health crises such as Haiti’s cholera epidemic, where Twitter was used as a data tool for immediately evolving circumstances. As such, we are confident in the thoroughness and accuracy of our findings.

Finally, we think that the critical implications of our findings include emphasizing the importance of using Twitter as a supplement to more conventional public health surveillance infrastructures. Twitter can potentially fortify researchers’ ability to collect data in a timely way [65,66] and improve the early identification of potential health threats [67]. In addition, as Twitter expands rapidly worldwide, results from Twitter-based studies can inform health policies related to social media, health communication, and prevention. More strategies regarding physical and mental health promotion that help people connect with reliable information and resources are necessary, and health promotion messages should be reframed to help users feel more comfortable accessing or reaching out to services related to mental health. Finally, public health agencies must continue to use Twitter and other social media platforms (eg, Facebook) as a new form of communication targeting audiences and strengthening health and prevention messages from more traditional media outlets (eg, television, radio, and print) [68-72]. Twitter can concurrently empower the fast and constant capture of the public’s perceptions, notions, and knowledge about health-related topics. It is an inexpensive development for communicating health campaigns and messages. Moreover, Twitter exhibits a considerable potential to modify messages and connect with people in real-time discussions about health and prevention.

### Strengths and Limitations

There are several strengths and limitations to this scoping review. First, compared with most review articles examining the benefits of using Twitter as a public health data source, this scoping review explicitly elaborates on the statistical language analyses and techniques implemented and used to study human health and behavior. Another strength is that this review provides a narrower objective than previous review papers by focusing on Twitter’s ability to surveil leading causes of mortality related to emerging disease epidemics, chronic diseases, and risk behaviors, representing 3 categories (respiratory infections, cardiovascular disease, and COVID-19). Finally, we incorporated studies that used Twitter to understand mental health conditions as mental health problems can increase the risk of chronic diseases and risky behaviors. The primary limitation of this scoping review is the restrictions in the search methodology. For example, excluding non–peer-reviewed literature, non–English-language articles, and works in progress and the focus on broader search strings used to identify literature may have prevented relevant studies from being discovered. Despite this limitation, this is common in scoping review studies as they are deliberate to broadly map a topic of interest while successfully achieving a balance between breadth and depth in a prompt time frame [73].

### Gaps and Future Research

Despite the existing literature on the potential application of Twitter in various public health settings, a limited amount of public health research has examined the relationships between users’ generated images and videos, specifically food-related and health-related behaviors. Most of the presented literature exclusively focused on using text to analyze and communicate. Visual content (ie, images and videos) has increasingly become the most shared content on social media platforms and may provide new avenues to study social and health-related behaviors. Food is one of the most common visual pieces of content shared on all social media platforms. There has been a strong association between the food people eat and how it affects people’s moods and feelings in many ways [74]. Previous research has found that people eat to relieve stress, depression, anxiety, and other harmful emotional strains [75,76]. To explore this gap, creating a computer-based process that simultaneously analyzes food-related images and text is needed. New techniques may provide insights into how food-related images can determine a positive or negative emotional state.

### Conclusions

As an emerging field, Twitter analysis has a promising future in public health communication and surveillance based on the 38 research articles included in this scoping review. Our findings shed light on the benefits of mining Twitter, provide evidence of language analysis applications that have been used to study tweets that can help identify subtle signals in language for understanding physical and mental health conditions and monitor emerging disease outbreaks, and could enable public health researchers interested in big data methodologies related to health outcomes to identify relevant Twitter-based studies. Most notably, our work extends the literature to include focusing on novel applications of language analyses and comprehensively reviewing public health surveillance of leading causes of mortality. In addition, beyond understanding and monitoring human health and behavior, our review shows that the Twitter platform can support public health policy and communication, thus being used to convey a broad scope of time-sensitive health and mental health promotion and prevention information. This innovation will conceivably expand the reach to hard-to-reach populations such as adolescents, who are more inclined to use and interact with Twitter than with more traditional public health media channels. Public health agencies nationwide are critical actors in this domain. It is enlightening to analyze how these agencies use relevant Twitter applications, which probably improve intelligence in communicating with their followers.

## Abbreviations

ADHD: attention-deficit/hyperactivity disorder
PRISMA: Preferred Reporting Items for Systematic Reviews and Meta-Analyses
PTSD: posttraumatic stress disorder
